# Differential Impact of Simultaneous or Sequential Coinfections With *Borrelia afzelii* and Tick-Borne Encephalitis Virus on the *Ixodes ricinus* Microbiota

**DOI:** 10.1155/ijm/7747795

**Published:** 2025-06-21

**Authors:** Apolline Maitre, Myriam Kratou, Ana Laura Cano-Argüelles, Stefania Porcelli, Lianet Abuin-Denis, Elianne Piloto-Sardiñas, Lourdes Mateos-Hernández, Dasiel Obregon, Miray Tonk-Rügen, Salma Kaoutar Abdelali, Sara Moutailler, Alejandro Cabezas-Cruz

**Affiliations:** ^1^ANSES, INRAE, Ecole Nationale Vétérinaire d'Alfort, UMR BIPAR, Laboratoire de Santé Animale, Maisons-Alfort, France; ^2^INRAE, UR 0045 Laboratoire de Recherches sur le Développement de l'Elevage (SELMET-LRDE), Corte, France; ^3^EA 7310, Laboratoire de Virologie, Université de Corse, Corte, France; ^4^Laboratory of Microbiology, National School of Veterinary Medicine of Sidi Thabet, University of Manouba, Manouba, Tunisia; ^5^Parasitology Laboratory, Institute of Natural Resources and Agrobiology of Salamanca (IRNASA, CSIC), Salamanca, Spain; ^6^Animal Biotechnology Department, Center for Genetic Engineering and Biotechnology, Havana, Cuba; ^7^Direction of Animal Health, National Center for Animal and Plant Health, Carretera de Tapaste y Autopista Nacional, San José de las Lajas, Cuba; ^8^School of Environmental Sciences, University of Guelph, Guelph, Ontario, Canada; ^9^Institute for Insect Biotechnology, Justus Liebig University, Giessen, Germany; ^10^Laboratory of Research on the Improvement and Development of Animal and Plant Production, University of Ferhat Abbas, Setif, Algeria

## Abstract

Ticks, particularly *Ixodes ricinus*, are significant vectors of pathogens such as *Borrelia* spp. and tick-borne encephalitis virus (TBEV), which cause Lyme borreliosis (LB) and tick-borne encephalitis (TBE), respectively. Understanding how these pathogens interact within the tick microbiome is essential for developing vector control strategies. This study investigates the impact of *Borrelia afzelii* and TBEV, as well as their coinfection, on the microbiota composition and structure of *I. ricinus* nymphs. Using a network-based approach, we analyzed the microbial communities of ticks exposed to infected or coinfected mice. DNA extracted from newly molted nymphs was sequenced for the bacterial 16S rRNA gene, and microbial diversity metrics (alpha and beta diversity) were calculated. Our results showed that TBEV infection increased microbiome diversity compared to the uninfected and *Borrelia* groups. Co-occurrence network analyses revealed that while microbial structures remained consistent across conditions, TBEV-infected networks exhibited higher robustness to perturbations, indicating a stabilizing effect on the tick microbiome. Furthermore, the hierarchical position and associations of *Borrelia* varied significantly depending on the infection scenario, highlighting its adaptive role within the tick microbiota. The study demonstrates that pathogen presence alters tick microbial dynamics, with TBEV enhancing stability, suggesting virus-mediated modifications of the microbiome. These findings advance our understanding of pathogen–tick–microbiome interactions and provide insights into the ecological mechanisms underlying pathogen coexistence within ticks. This research underscores the importance of microbial networks in ticks and offers new perspectives for targeted approaches in managing tick-borne diseases.

## 1. Introduction

Ticks are blood-sucking arthropods globally recognized as vectors of numerous pathogens affecting humans, domestic animals, and wildlife [1]. These ectoparasites belong to the class Arachnida and superorder Parasitiformes, with over 900 species divided into two main families: Ixodidae (hard ticks) and Argasidae (soft ticks) [2]. Among the around 700 hard tick species, *Ixodes ricinus* is notably abundant and the most common tick species in Europe [3], infesting birds, mammals, and occasionally reptiles [4, 5]. This tick exhibits a biphasic, seasonal activity pattern [6] and undergoes three blood feeding stages: larva, nymph, and adult. Each stage takes a single blood meal before molting to the next stage, or, in the case of an adult female, laying eggs. Adult males feed occasionally in small amounts [7, 8].

Lyme borreliosis (LB) is the most common vector-borne disease in the Northern Hemisphere [9]. Hard ticks of the genus *Ixodes* are the main vectors of Lyme pathogens and can also transmit *Borrelia miyamotoi*, the causative agent of relapsing fever (RF) borreliosis [10]. LB is a multisystemic inflammatory disease caused by spirochetes of *Borrelia burgdorferi* sensu lato (s.l.) complex, which includes at least 18 species [11]. In Europe, eight species from this complex have been reported: *Borrelia afzelii*, *Borrelia garinii*, *B. burgdorferi* sensu stricto (s.s.), *Borrelia valaisiana*, *Borrelia lusitaniae*, *Borrelia spielmanii*, *Borrelia bavariensis*, and *Borrelia bissettii*. Depending on the geographical location, the most common genospecies in *I. ricinus* are *B. afzelii* and *B. garinii* [11, 12]. Different animal groups vary in suitability as hosts for different life stages of *I. ricinus* [13, 14] and as reservoirs for different *Borrelia* species [15, 16]. For instance, in Europe, *B. afzelii* is associated with small mammals such as mice (*Apodemus* species) and the bank vole (*Myodes glareolus*), while *B. garinii* is associated with birds [17–19]. Previous studies have shown that *B. afzelii* establishes chronic infections in its rodent hosts that can last for months or even years [20]. *Borrelia* spirochetes are typically acquired by larval or nymphal ticks feeding on an infected vertebrate host [19]. Once ingested, the spirochetes colonize the tick's gut. During the tick's next blood meal, *Borrelia* migrates from the gut to the salivary glands and is transmitted to a new host via tick saliva by nymphs or adults [17, 21].

*I. ricinus* ticks, known carriers of various pathogens, also harbor viruses that cause significant medical and veterinary conditions [22]. One prominent pathogen among these is the tick-borne encephalitis virus (TBEV), *Orthoflavivirus encephalitidis*, which causes the zoonotic disease tick-borne encephalitis (TBE). This virus belongs to the tick-borne flavivirus group within the family Flaviviridae, genus *Orthoflavivirus* [23]. TBEV is the most important tick-transmitted arbovirus affecting humans in Europe and Asia, primarily transmitted by *I. ricinus* and *Ixodes persulcatus* [24]. In Western Europe, *I. ricinus* serves as the principal vector for TBEV [25]. Larvae of this tick species are typically uninfected due to the rarity of transovarial (vertical) transmission of TBEV [26, 27]. Instead, larvae or nymphs acquire the virus during a blood meal and maintain the pathogen after molting into the nymphal or adult stages through transstadial transmission. Once infected, ticks carry the virus for their entire life cycle [28].

Tick microbiota includes mutualist symbionts, commensal microorganisms, and pathogens affecting humans and animals [29, 30]. Ticks can acquire multiple pathogens through a single blood meal from a coinfected host or by feeding on different infected hosts during their sequential life stages [31–34]. When tick larvae and nymphs feed on an infected small mammalian host, they can ingest one or more pathogens, which may be transmitted during subsequent blood meals. The mechanisms underlying these coinfection patterns remain unclear, whether due to similar environmental preferences of pathogens, parallel acquisition from host communities, or direct microbe–microbe interactions within ticks [29]. Additional studies in North America have reported coexisting tick-borne pathogens among mammalian hosts. For example, in southern Connecticut, studies found that up to 50% of white-footed mice (*Peromyscus leucopus*) exhibited concurrent infection with *B. burgdorferi* s.s., *Babesia microti*, and *Anaplasma phagocytophilum*, highlighting the prevalence of coinfections in these reservoir hosts [35, 36]. Pathogenic bacteria transmitted by *Ixodes* ticks, such as *Rickettsia helvetica* and *B. burgdorferi* s.s., have been shown to modulate the tick microbiota, potentially facilitating their colonization and persistence [37, 38]. Mechanistically, colonization of *Ixodes scapularis* by *B. burgdorferi* s.s. induces the expression of a tick-secreted gut protein, PIXR, which suppresses biofilm formation by the gut microbiota. This reshaping of the gut environment promotes bacterial persistence in the tick midgut [38]. Not only do coinfections present diagnostic challenges, but also they can involve synergistic, antagonistic, or neutral interactions among pathogens within their hosts, thereby modulating disease severity [39–41]. Moreover, microbial interactions within the tick can influence pathogen establishment, persistence, and potential coexistence by shaping competitive or facilitative dynamics in the shared ecological niche [40, 41]. However, it remains largely unknown how coinfections influence the structure and function of the tick microbiota.

In this study, we examined how infection of *I. ricinus* larvae with *B. afzelii*, TBEV, or both pathogens influences the microbial community structure of the resulting nymphs. Larvae acquired these pathogens by feeding on C3H/HeN (C3H) mice that had been experimentally infected or coinfected in the study by Porcelli et al. [42]. We used newly molted nymphs derived from that experiment to investigate microbiome responses to distinct pathogen exposure scenarios. A network-based analytical framework was applied to assess structural and compositional changes in the tick microbiome. In order to evaluate the influence of these pathogen infections on the ‘core bacterial microbiota' and network properties, we performed an *in silico* removal of nodes from the networks, simulating the reshaping of bacterial community assembly in *I. ricinus* nymphs. This work underscores the ecological complexity of pathogen–microbiota interactions in ticks and provides insights relevant to vector competence and pathogen transmission control strategies.

## 2. Materials and Methods

### 2.1. Experimental Design

To investigate the effects of single and coinfections with *B. afzelii* and TBEV on the microbiota of *I. ricinus* nymphs, we analyzed newly molted nymphs derived from larvae that had fed on infected or coinfected C3H mice in the experimental study conducted by Porcelli et al. [42].

**Figure 1:**
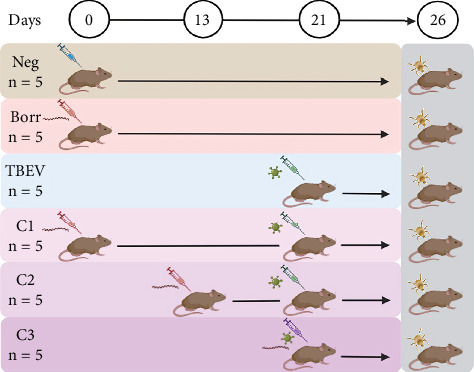
Experimental design of mice infection and coinfection. Mice (*n* = 5) of the negative (neg) group were inoculated with DMEM at Day (d) 0. For infection timing, we established the following groups: (1) *Borrelia* (Borr) group received *Borrelia afzelii* spirochetes on Day 0; (2) TBEV group received TBEV on Day 21; (3) coinfection groups received both pathogens with varying *B. afzelii* inoculation timing: coinfection 1 (C1) received *B. afzelii* on Day 0, coinfection 2 (C2) on Day 13, and coinfection 3 (C3) on Day 21. All coinfection groups (C1, C2, and C3) received TBEV on Day 21. On Day 26, *Ixodes ricinus* larvae were allowed to feed on the mice, and a subset was left to molt. Newly molted nymphs were collected and frozen at −80°C until further use.

Previously, six-week-old female C3H mice (Charles River Laboratories, France) were allocated into six experimental groups in the study by Porcelli et al. [42]. A negative control group (Neg group, *n* = 5) received Dulbecco's modified Eagle medium (DMEM) on Day (d) 0. Two single infection groups were also established: the Borr group (*n* = 5) was inoculated with *B. afzelii* on d0, while the TBEV group (*n* = 5) was inoculated with TBEV on d21 [42]. Three coinfection groups were set up as follows: *B. afzelii* was inoculated on d0 for the C1 group (*n* = 5), on d13 for the C2 group (*n* = 5), and on d21 for the C3 group (*n* = 5), followed by a TBEV inoculation on d21 for all coinfection groups (Figure 1; [42]). Mice were inoculated with 1 × 10^6^ spirochetes of *B. afzelii* CB43 strain in BSK-M medium, administered both subcutaneously (100 *μ*L) and intraperitoneally (150 *μ*L). For TBEV infection, the Hypr strain was delivered subcutaneously in a 100-*μ*L suspension containing 1 × 10^2^ plaque-forming units (PFU) per mouse. On d26, *I. ricinus* larvae were allowed to feed on the mice (two mice from each of the six groups). The larvae were obtained from a colony of the institute of Zoology, Slovak Academic of Sciences, Bratislava Slovakia. Upon engorgement, fully engorged larvae dropped and a batch of larvae from each experimental group were stored in an incubator at 22°C with 95% relative humidity and a 12-h light/dark cycle until molting. Following, 5–10 nymphs from each group were stored at −80°C in a freezer until further use [42]. To measure the presence of the pathogens in nymphs, a reverse transcription-preamplification-digital PCR was used to detect *B. afzelii* and TBEV RNA [42]. For our analysis, DNA samples were obtained from the newly molted *I. ricinus* nymphs that had fed as larvae on C3H mice in the previous study [42].

### 2.2. DNA Extraction and Reagent Controls

Genomic DNA (gDNA) was extracted from newly molted *I. ricinus* nymphs, which had previously fed as larvae on C3H mice [42]. Prior to DNA extraction, each tick was individually homogenized using the Fast Prep-24 5G homogenizer (MP Biomedicals, Irvine, CA, United States) with six stainless steel beads, in 200 *μ*L of DMEM supplemented with 10% fetal bovine serum (FBS). The homogenization process consisted of two cycles at 5500 rpm for 20 s each; then, samples were centrifuged at 1500 × g for 5 min. DNA was extracted from the supernatant using the NucleoSpin Tissue DNA Extraction Kit (Macherey-Nagel, Hoerdt, France), eluted in 30 *μ*L of DNase-free water, and stored at −80°C for future use. The concentration and quality of the extracted gDNA were determined using the NanoDrop One spectrophotometer (Thermo Scientific, Waltham, MA, United States), targeting an OD_260_/OD_280_ ratio between 1.8 and 2.0. Four reagent controls were also processed following the same procedures to monitor for any potential contamination, with these controls subjected to identical DNA amplification steps as the experimental samples.

### 2.3. 16S rRNA Sequencing and Processing of Raw Sequences

Genomic DNA extracted from nymphs (≥200 ng total, ≥20 ng/*μ*L) was sent for bacterial 16S rRNA gene amplicon sequencing, which was commissioned to Novogene Bioinformatics Technology Co. (London, United Kingdom). A single lane of the Illumina MiSeq system was used to generate 251-base paired-end reads from the V4 variable region of the 16S rRNA gene using bar-coded universal primers (515F/806R) in Neg (*n* = 12), Borr (*n* = 11), TBEV (*n* = 12), C1 (*n* = 12), C2 (*n* = 12), and C3 (*n* = 12) as well as extraction reagent controls (*n* = 4). The 16S rRNA sequences were analyzed using the Quantitative Insights Into Microbial Ecology 2 (QIIME2) pipeline (v.2022.8) [43]. The demultiplexed raw sequences (obtained in FASTQ files) were denoised, quality trimmed, and merged using DADA2 software [44] implemented in QIIME2 [43]. The reads were then merged, chimeric variants were removed, and taxonomically assigned using a pretrained naïve Bayes taxonomic classifier [45] based on the SILVA database (V. 138) [46]. The resulting taxonomic table was collapsed at the genus level and filtered by removing taxa with less than 10 reads and present in less than 30% of samples. The raw 16S rRNA sequences obtained from tick samples were deposited at the SRA repository (Bioproject No. PRJNA1123907).

### 2.4. Diversity Indexes and Taxa Abundance

We measured alpha diversity to characterize the microbial community within individual samples. Our alpha diversity analysis included metrics for evenness (species representation), phylogenetic diversity, and richness (number of taxa) [47]. The metrics were estimated using the q2-diversity plugin in QIIME2 environment. The alpha diversity evenness was explored using the Pielou's evenness index [48], the phylogenetic diversity (PD) with Faith's PD [49], and the richness with observed features [50]. Differences in the alpha diversity metric between groups were assessed using the pairwise Kruskal–Wallis test (*p* < 0.05) with QIIME2 and visualized using GraphPad Prism Version 9.0.2 (GraphPad Software, San Diego, California United States).

Beta diversity is a measure of diversity between conditions that assesses the similarity of microbial communities. Bacterial beta diversity was assessed using the Bray–Curtis dissimilarity index [51] and visualized in a principal coordinate analysis (PCoA) graph, with the Jaccard distance [52] test, and with the correspondence analysis of principal coordinates (CAP) [53] test. Bray–Curtis and Jaccard distance tests were analyzed with q2-diversity plugin in QIIME2 environment and compared between groups using the PERMANOVA test (*p* < 0.05). The CAP test was analyzed using the ‘*Vegan*' package [54], in RStudio [55], and a PERMANOVA test was conducted with the ‘*Adonis*' function [56]. Bray–Curtis, CAP, and Jaccard distance tests were visualized with ‘*Vegan*' package [54] implemented in RStudio [55]. Additionally, beta dispersion, a measure of sample variability within a condition, was calculated and analyzed using ANOVA (*p* < 0.05) with the ‘*Vegan*' package in RStudio [55].

The difference of taxa composition was assessed with an UpSet graph to visualize which taxa are common between the different conditions. This graph was constructed with the ‘*UpSetR*' package [57] in RStudio [55]. The taxa abundance differences were assessed to measure statistically different bacterial abundance between the groups. The abundance of taxa was transformed to centered log ratio (clr) value, with ALDEx2 package [58] implemented in RStudio [55]. The statistically different taxa (Kruskal–Wallis test, *p* < 0.05) were used for representation of the taxa differential abundance. The resulting data were used to construct the heatmap with the ‘*heatmap.2*' function from gplots package [59] implemented in RStudio environment [55].

### 2.5. Bacterial Co-Occurrence Network Construction

Microbial structure and organization were analyzed by co-occurrence network analysis. The networks allow the graphic visualization of the microbial community assemblies. Bacterial taxa are represented by nodes and the significant correlations between taxa are represented by edges. Analyses of significant positive (weight > 0.5) or negative (weight < −0.5) correlations were performed using the SparCC method [60] implemented in RStudio environment [55]. For this analysis, network interactions were calculated with a bootstrap of 1000 and interactions above the threshold of 0.05 were removed. The visualization of the networks was performed using the software Gephi 0.10 [61]. Network topological features were calculated: number of connected nodes and edges (positive and negative), modularity (the strength of the division of a network into modules), number of modules, network diameter (the shortest path between the two most separated nodes), average shortest path, average degree (the average number of links per nodes), weighted degree (the sum of the weight of all the edges connected to a node), and clustering coefficient (the degree to which nodes in a network tend to form clusters).

### 2.6. Robustness Analysis

The robustness of the microbial networks to perturbation (infection or coinfection) in each experimental group was evaluated using two techniques: loss of connectivity in response to node removal and network resilience to node addition. For node removal analysis, nodes were removed in four ways: high betweenness centrality (frequency with which a node is situated on the shortest path between pairs of other nodes), cascading (high betweenness centrality recalculated when each node is removed), high degree (number of edges that a node has with other nodes), and random removal. The loss of connectivity (IC 95%) was calculated depending on the node removal rate and compared between the conditions. This was performed using Network Strengths and Weaknesses Analysis (NetSwan) script [62] in RStudio [55].

The node addition consists of new nodes randomly selected and connected to the existing network. To this end, we determined the size of the largest connected component (LCC) (largest subset of nodes that are mutually reachable through edges) and the average path length (APL) (average number of steps needed to travel between any two nodes in the network). The LCC and APL are then calculated every 10 nodes added, from 10 to 100. The simulation was repeated 10 times for more strength of analysis. This analysis was described by Freitas et al. [63] and performed with RStudio [55]. The obtained values were plotted using GraphPad Prism 9.0.2 (GraphPad Software, Boston, Massachusetts United States) to visualize the results.

### 2.7. Network Comparison

To compare the networks' most central nodes, the Jaccard index was calculated for degree, betweenness centrality, closeness centrality, eigenvector centrality, and hub taxa. The Jaccard index tests the similarity between sets of “most central nodes” of networks, which are defined as those nodes with a centrality value above the empirical 75% quartile. Thus, this index expresses the similarity of the sets of most central nodes as well as the sets of hub taxa between the two networks. Jaccard index ranges from 0 (completely different sets) to 1 (sets equal). The two *p* values *p*(*J* ≤ *j*) and *p*(*J* ≥ *j*) for each Jaccard's index are the probability that the observed value of Jaccard's index is ‘less than or equal' or ‘higher than or equal,' respectively, to the Jaccard value expected at random which is calculated taking into account the present total number of taxa in both sets [64]. In addition, the top 10 nodes with the highest degree, eigenvector centrality, and betweenness centrality were compared between conditions using Gephi v.0.10 [61]. The Adjusted Rand Index (ARI) was also calculated to test the dissimilarity of clustering in the networks. ARI values range from −1 to 1. Negative and positive ARI values mean lower and higher than random clustering, respectively. An ARI value of 1 corresponds to identical clustering, and 0 to dissimilar clustering. The *p* value tests if the calculated value is significantly different from zero [65]. Jaccard index and ARI were calculated between all pair of conditions (Neg-Borr, Neg-TBEV, Neg-C1, Neg-C2, Neg-C3, Borr-TBEV, Borr-C1, Borr-C2, Borr-C3, TBEV-C1, TBEV-C2, TBEV-C3, C1-C2, C1-C3, and C2-C3) with ‘*Netcomi*' package [65] implemented in RStudio [55]. A second network comparison was conducted with the core-associated network (CAN) analysis, which analyzes sets of nodes that are similarly connected between different networks [66]. The analysis was performed between all pairs of conditions with the anuran toolbox implemented in Python environment (https://github.com/ramellose/anuran).

The topology of the taxa in the networks was analyzed using a *P*_*i*_–*Z*_*i*_ test. This test calculates the within-module (*Z*_*i*_) and the among-module (*P*_*i*_) connectivity of nodes in a network. It ranges nodes in four categories: (i) peripheral taxa (*Z*_*i*_ ≤ 2.5 and *P*_*i*_ ≤ 0.62), which contain taxa with few edges in and out of their module; (ii) connectors (*Z*_*i*_ ≤ 2.5 and *P*_*i*_ > 0.62), which contain taxa connected to other modules than their own; (iii) module hubs (*Z*_*i*_ > 2.5 and *P*_*i*_ ≤ 0.62), which contain taxa highly connected with members of their own module; and (iv) network hubs (*Z*_*i*_ > 2.5 and *P*_*i*_ > 0.62), which contain taxa highly connected with members within and among their module. For each taxon, *Z*_*i*_ and *P*_*i*_ values were calculated using only positive edges, with the R script “code-zi-pi-plot” described by [67, 68] in RStudio [54], and visualized with GraphPad Prism Version 9.0.2 (GraphPad Software, Boston, Massachusetts United States).

### 2.8. *Borreliella* Hierarchical Position in the Microbiota

The position of the *Borreliella* genus (including *B. afzelii*) within the tick microbiota was evaluated to assess whether its presence, along with TBEV, influences its interactions and hierarchical position. To compare its position across conditions, the *Borreliella* node was analyzed within the co-occurrence network and its module. The hierarchical position of *Borreliella* node was further evaluated by analyzing its direct neighbors and calculating centrality measures (degree, betweenness centrality, clustering coefficient, and eigenvector centrality). These analyses were conducted using Gephi 0.10 [61].

## 3. Results

### 3.1. Raw Sequence Quality and Abundance of Taxa Per Reads

After the filtering, denoising, merging, and chimeric removal, our analyzed reads per sample ranged from 29,016 reads, representing 44.53% of the raw number of reads, to 273,783 reads, representing 83.18% of the raw number of reads (Ave ± = 75,187.1 ± 49,154.12 reads representing 78.89 ± 7.90% of the raw number of reads; Table S1). A total of 204 taxa were identified with the number of reads ranging from 12 (Ave ± = 0.17 ± 1) for *Solirubrobacter* to 916,448 (Ave ± 13,092.11 ± 17,170.22) for *Candidatus* Midichloria (Table S1).

### 3.2. TBEV Presence Increases Microbial Alpha Diversity

To examine how infection with *B. afzelii* alone, TBEV alone, or coinfection with both pathogens affects *I. ricinus* microbiome, we sequenced the 16S rRNA gene from genomic DNA of newly molted nymphs. These nymphs had previously fed as larvae on either uninfected mice or mice infected with *B. afzelii*, TBEV, or both pathogens [42]. After sequencing, the four extractions reagent controls did not contain microbial sequences and did not meet the threshold for 16S rRNA amplicon sequencing, confirming that these samples were free from contamination.

**Figure 2:**
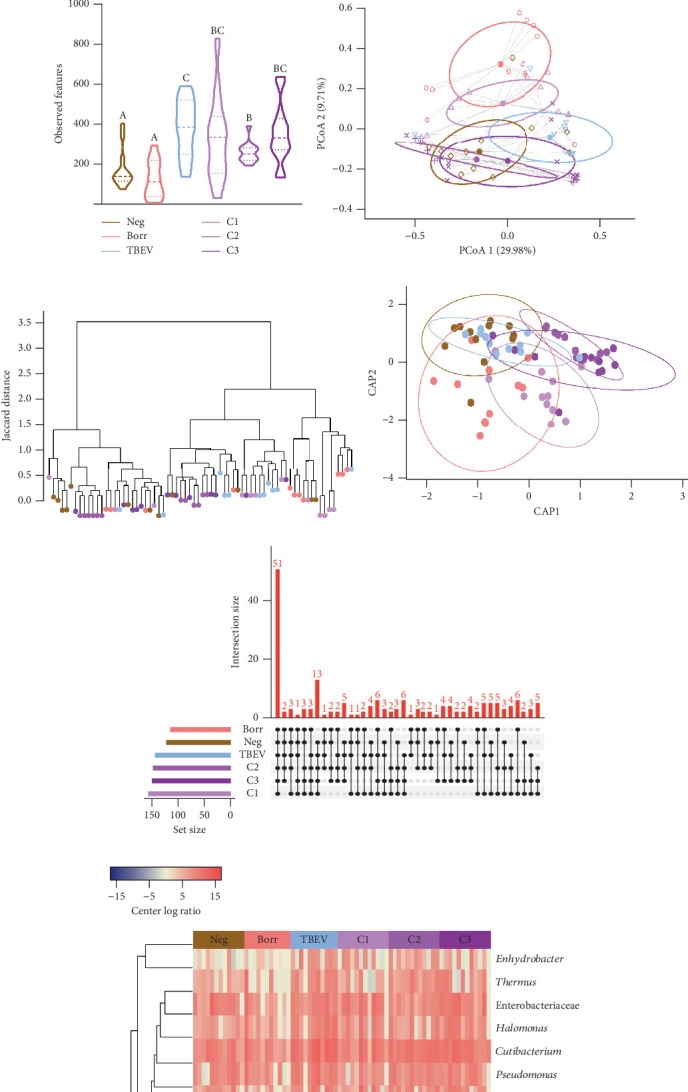
Bacterial diversity and abundance comparison of the tick microbiota. The alpha diversity metrics: (a) Pielou's evenness, (b) Faith's PD and (c) observed features were compared between the groups (control, Borr, TBEV, C1, C2, and C3). The same letter means no significant differences whereas different letters mean significant differences (paired Kruskal–Wallis test, *p* < 0.05). The beta diversity metrics: (d) Bray–Curtis, (e) Jaccard distance, and (f) CAP were compared between the groups (control, Borr, TBEV, C1, C2, and C3). Bray–Curtis and Jaccard distance statistical test results are presented in Table S2. The CAP analysis was found significant by the PERMANOVA test (*p* < 0.001). (g) The UpSet graph represents bacterial taxa common and unique to each condition (control, Borr, TBEV, C1, C2, and C3). (h) Heatmap represents bacterial taxa significantly different between conditions (control, Borr, TBEV, C1, C2, and C3) at the Kruskal-Wallis test (*p* <0.05), Asterisk denotes taxa significantly different between conditions at the Kruskal–Wallis test with Benjamini–Hochberg correction (*p* < 0.05).

The alpha diversity metrics (Pielou's evenness, Faith's PD, and observed features) demonstrated similar results (Figures 2a, 2b, and 2c), with a significantly less rich and even microbiota for Borr and Neg compared to the TBEV group (pairwise Kruskal–Wallis, *p* < 0.05). Beta diversity metrics demonstrated significant differences between all the conditions except C1 versus C3 and C2 versus C3 for Bray–Curtis test and C2 versus C3 and C3 versus TBEV for Jaccard distance (PERMANOVA, *p* < 0.05; Table S2; Figure 2d,e). These results suggests that the time of injection of *B. afzelii* in mice may significantly impact the diversity of microbial communities in *I. ricinus* nymphs. The CAP test demonstrated significant differences between the different conditions (PERMANOVA, *p* < 0.001; Figure 2f). In the comparison of the microbial composition, the six conditions shared 51 of 179 taxa (28% of common taxa, Figure 2g) and 13 of 179 taxa (7%) were shared between all the groups except Borr (Figure 2g). The differential abundance test measured 13 taxa with different clr values between the groups (Kruskal–Wallis, *p* < 0.05, Figure 2h), including *Borreliella* that was more abundant in the Borr and C1 groups.

### 3.3. TBEV Presence Increases Network Robustness

In this study, we also explored the influence of TBEV and *B. afzelii* infections, as well as their coinfection, on the microbial community assembly of *I. ricinus* nymphs. To this end, we built the bacterial co-occurrence networks and tested their resilience to perturbations, such as addition and removal of nodes.

**Figure 3:**
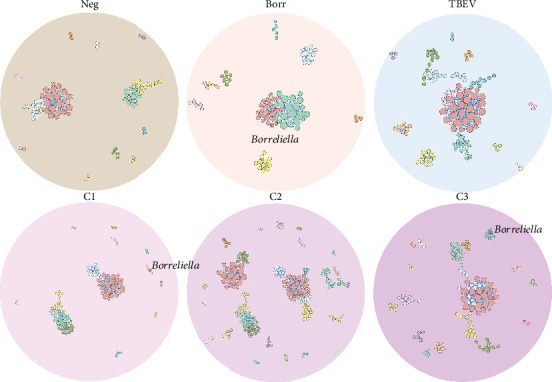
Network comparison of the tick microbiota. Representation of the microbial networks of the six conditions. Nodes represent a bacterial taxon, edges represent a co-occurrence interaction, positive in blue (edge weight > 0.5) and negative in red (edge weight < −0.5). In each network, the same node color indicates the same module, and the node size represents the eigenvector centrality value. The taxa *Borreliella* is represented by an asterisk.

Visually, the microbial networks presented similarities, with the number of connected nodes ranging from 81 (Neg) to 128 (C3) and edges varying from 179 (Neg) to 405 (TBEV), essentially positives (Table S3; Figure 3). It is noteworthy that all networks exhibited positive correlations between central modules and peripheral modules, with the latter comprising fewer than 10 bacterial taxa. This observation suggests that the infection or coinfection did not significantly alter the association of taxa within the microbial community. Additionally, networks exhibited a similar modularity value (Ave = 0.71 ± 0.05) and a number of communities proportional to the networks size (Table S3; Figure 3).

**Figure 4:**
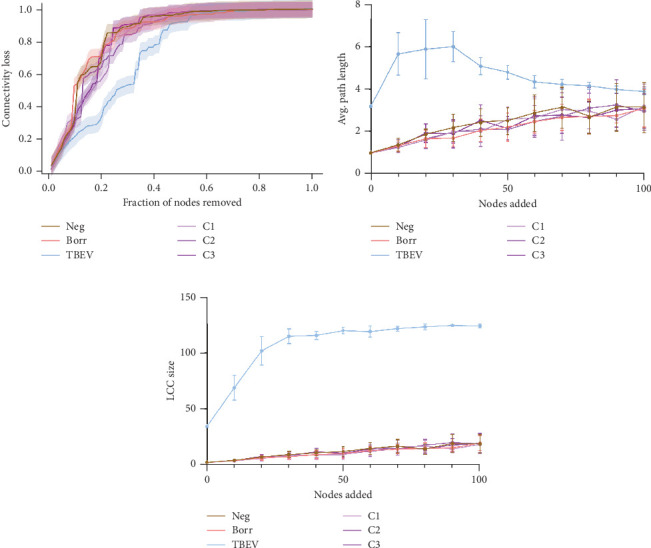
Robustness comparison of the tick microbial networks. Robustness of the microbial networks was assessed with (a) the effect of node removal on the connectivity loss. The colored line represents the connectivity loss depending on removing nodes with high degree first. The confidence interval is represented by the colored zone following the line. Robustness was also assessed with the effect of node addition on (b) the average (avg.) path length and (c) the largest connected component (LCC) size. Zero to 100 nodes were added to the networks. The line represents avg. path length or LCC size depending on the number of nodes added; the confidence interval is represented at each 10 nodes.

The robustness tests demonstrated a clear shift between the TBEV group and the rest of the conditions. The node removal revealed that TBEV was more robust to degree attack (Figure 4a) and to betweenness (Figure S1), notably. Regarding the node addition metrics, the TBEV group exhibited a higher LCC than the other groups when 0 and 30 nodes were added. Subsequently, its LCC values decreased to similar levels to those of the other groups (Figure 4b). These findings indicate that the TBEV network is more resilient to disruptions than the other groups. However, the APL was higher for the TBEV group compared with the other conditions indicating inefficiencies in the communication between the nodes.

### 3.4. Microbial Networks' Hierarchical Composition Varies Depending on Pathogen Coinfection

Next, a comparison was conducted between the networks based on the terms of the distribution of local centrality measures and clustering. The comparison of the most central nodes with the Jaccard index demonstrated that the central nodes exhibited the greatest similarity between C1-C2 for degree (Jacc = 0.32), C1-C3 for betweenness centrality (Jacc = 0.38), Borr-C2 and C1-C2 for closeness centrality (Jacc = 0.34), and C2-Neg for eigenvector centrality (Jacc = 0.36) and for hub taxa (Jacc = 0.36; Table S4). Overall, none of the tested pairs were statistically equal to or more similar than random (*p* < Jacc; Table S4). The 10 top nodes of degree, eigenvector centrality, and betweenness centrality were compared, and the C1 and C2 groups have only *Dongia* as a common top node for degree and eigenvector centrality (Table S5). The C3 and TBEV groups had two common top nodes, *Pedomicrobium* and *Alloprevotella*, for degree and eigenvector centrality (Table S5). For the top betweenness centrality nodes, *Morganella* taxa were common between the Borr, C1, C2, and Neg groups demonstrating its importance in the network when TBEV is less present. Simultaneously, *Pseudoxanthomonas* appeared among the top nodes in the C3, Neg, and TBEV groups, highlighting its significance in the network when *Borreliella* is less present (Table S5).

The most dissimilar central nodes were between C3-Neg for degree (Jacc = 0.13, *p* (<Jacc) = 0), Borr-TBEV and C1-TBEV for betweenness centrality (Jacc = 0.23, *p* (<Jacc) < 0.05), C3-Neg for closeness centrality (Jacc = 0.17, *p* (<Jacc) = 0), eigenvector centrality (Jacc = 0.11, *p* (<Jacc) = 0), and for hub taxa (Jacc = 0.11, *p* (<Jacc) = 0; Table S4). Overall, at least one of the five centrality measures tested was statistically equal to or more different than random (*p* > Jacc) for Borr-C3, Borr-TBEV, C1-C3, C1-Neg, C1-TBEV, C2-C3, C2-TBEV, C3-Neg, and Neg-TBEV (Table S4). In those group comparisons, Borr-C3 and C1-C3 did not share any taxa among the top central nodes for degree, eigenvector centrality, and betweenness centrality (Table S5). Additionally, the ARI values ranged from 0.05 for C2-C3 and C1-TBEV (*p* < 0.05) to 0.25 for Borr-Neg (*p* = 0). The results of the Jaccard index and ARI demonstrated that the microbial networks did not exhibit similarities in the most central nodes or in the clustering, indicating a shift in the hierarchical organization caused by the pathogen infection or coinfection. Moreover, the CAN analysis demonstrated the greatest similarity between C1 and C2 (21 nodes and 14 edges), and the least similarity between Borr and TBEV (6 nodes and 5 edges; Figure S2).

**Figure 5:**
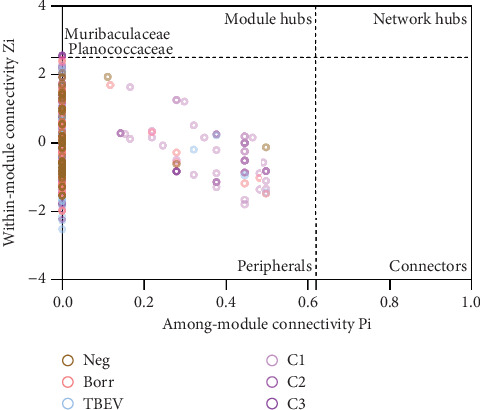
Connectivity plot of among-module and within-module interactions. The connectivity was measured based on two factors: among-module (*P*_*i*_) and within-module (*Z*_*i*_). The plot categorizes the taxa, represented by dots, into four distinct categories, represented by dash lines: peripherals (taxa located at the periphery of network), connectors (taxa that facilitate connections between different modules), module hubs (taxa with high connectivity within their respective modules), and network hubs (taxa that exhibit high connectivity across the entire network).

For the analysis of the taxa topology, all taxa were categorized into the peripheral group, except for Planococcaceae and Muribaculaceae in the C3 network, which were included in the module hub group (Figure 5). This could suggest a relevant role of these taxa within the C3 group microbiome.

### 3.5. *Borreliella* Hierarchical Position Varies Between Conditions

The position of the *Borreliella* taxon varied between the conditions (Figure 3). *Borreliella* node was absent in the Neg, TBEV, and C2 groups, but was in a major module in Borr network, along with 21 other taxa (Figure 3). Moreover, *Borreliella* taxon was in a peripheral module in C1 (with three other members) and C3 groups (with five other members). None of the module members of *Borreliella* were common between the conditions, demonstrating dissimilar role, or niche in the tick's microbiota (Table S6).

**Figure 6:**
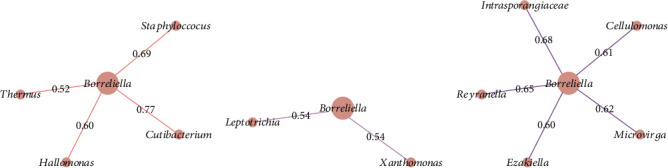
*Borreliella* reference networks. Networks representing direct neighbors *Borreliella* in the (a) Borr, (b) C1, and (c) C3 groups. The numbers represent the edge weight value. The same node color means the same module, and the node size reflects its eigenvector centrality value.

The co-occurrence networks revealed that all direct neighbors of *Borreliella* were all positive co-occurrence interactions for Borr (*Thermus*, *Cutibacterium*, *Halomonas*, and *Staphylococcus*, Figure 6a), C1 (*Leptotrichia* and *Xanthomonas*; Figure 6b), and C3 groups (Intrasporangiaceae, *Cellulomonas*, *Microvirga*, *Ezakiella*, and *Reyranella*; Figure 6c). *Borreliella* taxon was the most central node in the Borr group compared with the other conditions, with the highest eigenvector centrality value (*n* = 0.13; Table S7); it was also well connected with five microbial taxa in C3 (*n* = 5; Table S7).

## 4. Discussion

Recent surveys have reported a higher prevalence of coinfections, with approximately 50% or more of ticks harboring up to five different pathogens [69–73]. LB and TBE are caused by infections with *B. burgdorferi* s.l. and TBEV, respectively. In Europe, both pathogens are transmitted to humans through the bite of *I. ricinus*, which may also expose humans to other microorganisms carried by this tick, occasionally resulting in infection [74]. These coinfecting pathogens interact with host symbionts, influencing the utilization of host resources and modulating host immunity [40, 75]. Due to the high levels of coinfection in ticks and hosts, they serve as a useful model to study mechanisms of pathogen coexistence [40, 70, 71, 76]. To date, research on coinfections in ticks has primarily focused on studying the pathogens involved and the immune responses of both the host and the tick. Similarly, the influence of the tick microbiome on pathogen coinfection remains largely unexplored [77, 78]. In the present work, we have developed an innovative strategy to study dual infection in ticks from a microbial population perspective, using microbial co-occurrence networks.

In a previous study, Porcelli et al. [42] developed a model for investigating infections and coinfections with *B. afzelii* and TBEV in C3H mice. The findings of our research indicate that exposure of *I. ricinus* ticks to infected animals can influence the composition and dynamics of the microbial community. This was achieved by sequencing the 16S rRNA from nymphs molted from larvae fed on infected and coinfected mice. The infection with *B. afzelii* demonstrated a reduction in bacterial richness, with no significant differences observed when compared to the Neg group. This result contrasts with the findings reported by Hamilton et al. [79], where nymphs infected with *B. afzelii* exhibited a less abundant but more diverse bacterial community. Conversely, TBEV infection resulted in significant differences when compared to both the Neg and Borr groups, suggesting a more diverse bacterial community.

The co-occurrence network analysis revealed a similar structure in the community assembly, with positive associations between the taxa of *I. ricinus* microbiome in all conditions. Our results align with those of a previous analysis of *I. ricinus* nymphs collected in the field, in which the majority of observed interactions among taxa were positive [80]. Interestingly, the TBEV network exhibited greater resilience to the removal and addition of nodes. While higher diversity and stability are often correlated [81], these findings suggest that the virus exerts a stabilizing influence on the tick microbiome. TBEV infection has been observed to induce metabolic alterations in the host organism, particularly linked to inflammatory responses and immune modulation [82]. Nonetheless, the specific influence of tick-borne viruses on the tick microbiome remains largely uninvestigated [83].

Tick-borne pathogens coexist and interact with various bacterial species within the tick microbiome, forming an ecological unit referred to as the tick holobiont [84]. These interactions impact the vectorial capacity through a bidirectional relationship between the microbiota and pathogens [85]. This relationship may influence other bacteria, potentially explaining the observed shifts in *Borreliella* associations within the *I. ricinus* microbiome under different experimental conditions (Figure 6). Previous studies have also demonstrated a robust correlation between the presence of certain pathogens and the structure of the *I. ricinus* microbiota [80]. These findings lend support to the notion that pathogens need to alter microbial dynamics in order to establish themselves and maintain a persistent presence within the tick.

During viral and bacterial infections, the host organism responds to signaling molecules generated in response to the pathogen's presence [86, 87]. This response activates leukocytes, which increase the activity of prooxidative enzymes responsible for generating reactive oxygen species (ROS), crucial for the host's immune defense against pathogens [88]. For instance, coinfection with *A. phagocytophilum* and *B. burgdorferi* s.s. has been shown to result in shifts in inflammatory cytokines such as IL-12, TNF-*α*, and IFN-*γ*, as well as alterations in the antibody response to *A. phagocytophilum* in mice [33, 89]. In ticks, specifically *I. scapularis*, those fed on *B. burgdorferi*-infected mice have been observed to acquire host IFN-*γ*, which then activates the tick's Rho-like GTPase via STAT signaling, resulting in the expression of antimicrobial peptides (e.g., Dae2). This enables the tick to initiate defense mechanisms to control the proliferation of ingested pathogens from the blood meal [90]. It is plausible that this immune activation could also influence the broader microbiome within the tick. The production of ROS and antimicrobial peptides might create selective pressures, potentially modulating the tick's microbiota composition by impacting microbial survival. This modulation could favor certain symbiotic bacteria while limiting the proliferation of other strains, including opportunistic or pathogenic ones. Our findings, showing shifts in the *I. ricinus* microbial community structure after exposure to pathogens such as TBEV and *B. afzelii*, may reflect such immune-driven processes. Although the exact mechanisms remain to be fully elucidated, these interactions suggest a complex interplay where pathogen-induced immune responses could indirectly shape the tick microbiome, influencing both microbial diversity and pathogen persistence. Further studies are needed to unravel the extent and specific pathways through which the tick's immune system interacts with and modulates its microbiota.

Our sequence data confirmed the dominance of *Candidatus* Midichloria mitochondrii in *I. ricinus*: this endosymbiont accounted for >900 k read pairs—nearly an order of magnitude more than any other taxon. *Midichloria* is a vertically transmitted, intramitochondrial bacterium that has been detected in virtually every *I. ricinus* population examined to date and is considered part of the tick's “core” microbiome [91, 92]. Because Figure 2 depicts only those genera whose clr abundance differed significantly between experimental groups, Midichloria—though highly abundant—does not appear in the heat-map; its relative abundance remained statistically unchanged across uninfected, singly infected, and coinfected treatments, suggesting that neither *B. afzelii* nor TBEV perturbs this obligate symbiont. The stability of Midichloria is consistent with its proposed essential functions (e.g., provision of B vitamins and modulation of oxidative metabolism) and with reports that experimental suppression of Midichloria-like organisms can reduce tick fecundity [93].

By contrast, we did not detect *Wolbachia* above our filtering thresholds (≥30% of samples and ≥10 reads). Apparent *Wolbachia* signal in ticks is frequently attributable to DNA from the parasitoid wasp *Ixodiphagus hookeri* or other arthropod contaminants rather than true infection of the tick itself [94]. Similar *Wolbachia*-negative results have been obtained for both field and laboratory populations of *I. ricinus* and *I. scapularis* when rigorous contamination controls are applied [95]. Together, these findings highlight the importance of stringent quality filters and ecological context when interpreting low-abundance 16S rRNA signatures in tick microbiome studies.

## 5. Conclusions

This study demonstrates that *B. afzelii* and TBEV infections significantly impact the microbiome of *I. ricinus* ticks. TBEV infection was associated with increased microbiome diversity and network resilience, suggesting that the virus may stabilize microbial communities within ticks. In contrast, *B. afzelii* infection did not significantly alter microbiome diversity, indicating more specific effects on certain microbial taxa. Our findings reveal the complex dynamics between pathogens and the tick microbiome, highlighting that coinfection scenarios can modulate microbial community structure and influence vector competence. The study emphasizes the utility of network analysis in understanding microbial interactions and suggests that viral infections could enhance microbiome stability, potentially affecting pathogen persistence. Future research should explore the molecular mechanisms behind these interactions, as understanding the tick holobiont may inform novel strategies for controlling tick-borne diseases.

## Data Availability

The data that support the findings of this study are openly available in SRA at https://www.ncbi.nlm.nih.gov/sra/, reference number PRJNA1123907.

## Supporting Information

Supporting Information 1 Figure S1: Robustness of tick microbial networks per condition. Effect of node removal on the connectivity loss of each microbial conditions. The colored line represents the connectivity loss depending on removing nodes by random (blue), high betweenness (red), high degree (orange), or cascading (high betweenness recalculated at each removal) first. The confidence interval is represented by the colored zone following the line.

Supporting Information 2 Figure S2: Core-associated network and reference taxa comparison of the tick microbial networks. The core associated network (CAN) represents common co-occurrence interaction between the different groups (Control, Borr, TBEV, C1, C2, and C3). In each network, the same node color indicates the same module, and node size represents the eigenvector centrality value. Asterisk represents uncultured taxa.

Supporting Information 3 Table S1: Raw sequences quality and abundance of taxa per reads.

Supporting Information 4 Table S2: Statistical *p* values of Bray–Curtis and Jaccard index tests. *p* values between two conditions (Group 1 versus Group 2) are presented in bold when significant (*p* < 0.05) for Bray–Curtis and Jaccard index tests.

Supporting Information 5 Table S3: Network topological features. The number of connected nodes, connected edges (positives and negatives), modularity, number of modules, network diameter, shortest path, average degree, weighted degree, and clustering coefficient were measured for the conditions (control, Borr, TBEV, C1, C2, and C3).

Supporting Information 6 Table S4: Jaccard comparison and Adjusted Rand Index of the most central nodes between networks. Comparison between Condition 1 and Condition 2 of the Jaccard value (Jacc). *p* values of the comparison of most central nodes to be lower than (*p* < Jacc), or higher than (*p* > Jacc) random distribution. Significant value is represented with ⁣^∗^ (⁣^∗^*p* < 0.05 and ⁣^∗∗∗^*p* < 0.001).

Supporting Information 7 Table S5: Highest central nodes of each microbial network. Common nodes are represented in a red box.

Supporting Information 8 Table S6: Direct co-occurrence interaction of the *Borreliella* node in microbial networks.

Supporting Information 9 Table S7: Central measures of *Borreliella* in microbial networks.
