# Supplementary figures and images for: Differential Impact of Simultaneous or Sequential Coinfections With Borrelia afzelii and Tick-Borne Encephalitis Virus on the Ixodes ricinus Microbiota
Source: Int J Microbiol. 2025 Jun 21;2025:7747795. doi: 10.1155/ijm/7747795 (PMC12206002; doi:10.1155/ijm/7747795)

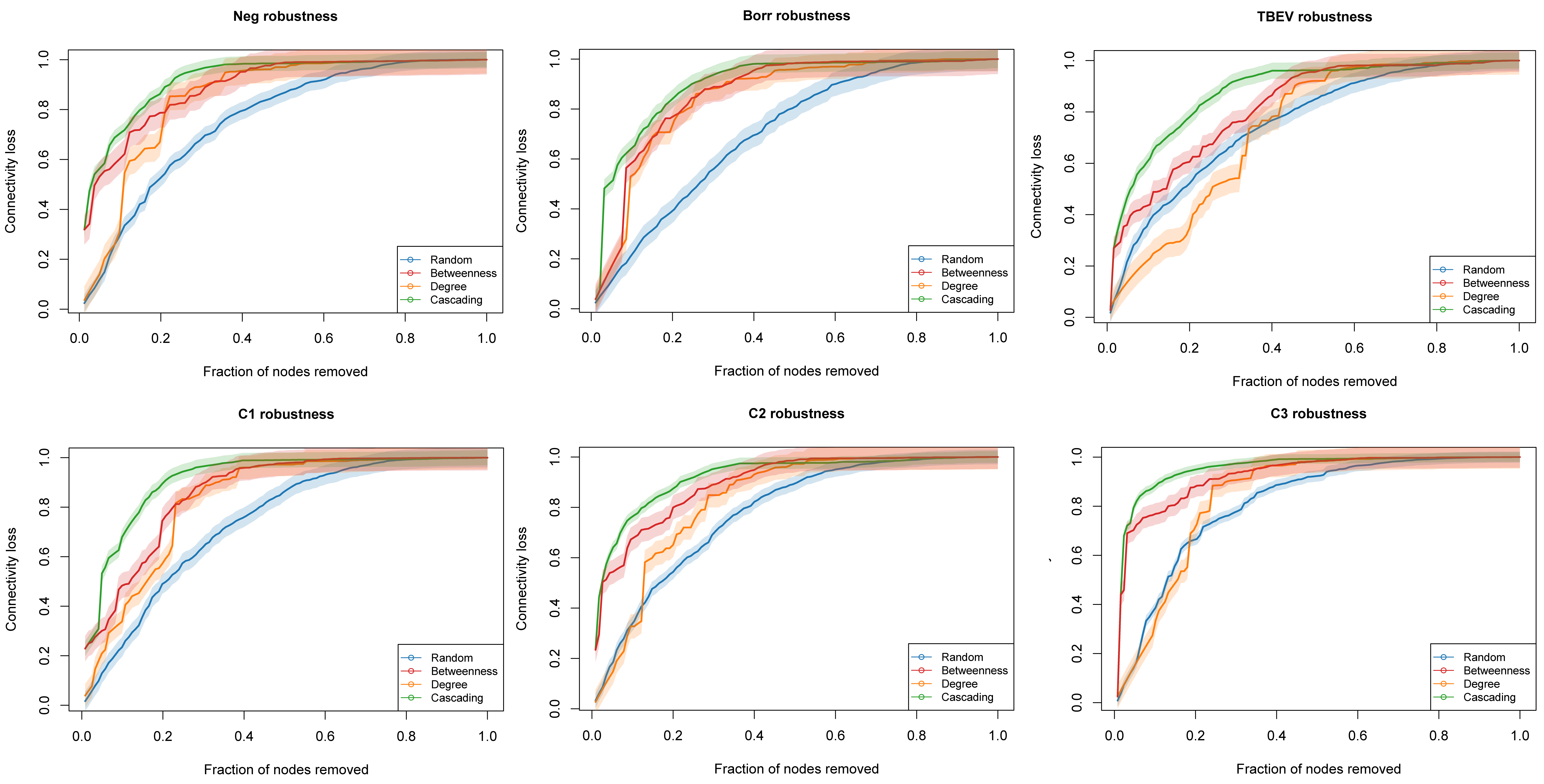

Supplement: Supporting Information 1 — Figure S1: Robustness of tick microbial networks per condition. Effect of node removal on the connectivity loss of each microbial conditions. The colored line represents the connectivity loss depending on removing nodes by random (blue), high betweenness (red), high degree (orange), or cascading (high betweenness recalculated at each removal) first. The confidence interval is represented by the colored zone following the line. [file 7747795.f1.png]
